# Atlanta Medical College Commencement, &c.

**Published:** 1889-04

**Authors:** 


THE ATLANTA MEDICAL COLLEGE COMMENCEMENT came off on the evening of the 4th of March. The annual oration was made by the Rev Dr Strickler, on the subject of Evolution. It was an able address and listened to with profound attention.

The valedictorian of the class was Dr J P Bowdoin. The following is a list of the graduating class: J P Bowdoin, P E Carr, J T Cobb, J C Embry, George C Erwin, A T Ford, Cicero Gibson, A J Gilbert, A S Gilbert, W C Hanson, H F Harris, J W Hewell, P M Hodgson, R L Hollis A S Howard, Paul Kendall, B M Kennon, M B McAfee, W L McBath, J J McEvoy, R D McLeod, L B McWhorter, H L Mobley, J W Neal, J M Neal, W L Patton, J W Peek, Paul E Penniston, Y L Pool, D D Quillian, J D Robinson, J L Simpson, Jas W Smith, T E Stokes, F W Storey, G W Strickland, C W Taylor, Scott Thomson, W W Tison, W J Warren, G W Westbrook, Willis Winn, E L Wright and I H Goss.

The prizes were as follows:

The first honor was awarded Dr Henry F Harris, of Georgia.

The second honor was won by Dr W C Hanson, of Alabama.

The third honor was awarded Dr J W Hewell, of South Carolina.

